# Developing a complex intervention for the outpatient management of incidentally diagnosed pulmonary embolism in cancer patients

**DOI:** 10.1186/1472-6963-13-235

**Published:** 2013-06-27

**Authors:** June Palmer, George Bozas, Andrew Stephens, Miriam Johnson, Ged Avery, Lorcan O’Toole, Anthony Maraveyas

**Affiliations:** 1 Queen’s Centre for Oncology and Haematology, Castle Hill Hospital, Cottingham, Hull HU16 5JQ, United Kingdom; 2 Department of Radiology, Castle Hill Hospital, Cottingham, Hull HU16 5JQ, United Kingdom; 3 Hull York Medical School, University of Hull, Hull HU6 7RX, United Kingdom

**Keywords:** Incidental pulmonary embolism, Outpatient management, Nurse-lead, Complex intervention

## Abstract

**Background:**

Most patients with pulmonary embolism (PE) spend 5–7 days in hospital even though only 4.5% will develop serious complications during this time. In particular, the group of patients with incidentally diagnosed PE (i-PE) includes many patients with low risk features potentially ideal for outpatient management; however the evidence for their optimal management is lacking hence relative practices may vary considerably. We describe the development process, components, links and function of a nurse-led service for the management of patients with i-PE, developed in accordance to the UK Medical Research Council complex intervention guidance.

**Methods:**

*Phase 0 (Theoretical underpinning):* The Pulmonary Embolism Severity Index (PESI) was selected for patient risk assessment and the American Society of Clinical Oncology (ASCO) guideline for the management of PE in cancer patients (2007) was selected as quality measure. Historical registry and audit data from our centre regarding i-PE incidence and management for the period between 2006 and 2009 illustrating the then current practices were reviewed. *Phase 1 (Modelling):* Modelling of the pathway included the following: a) Identification of training needs, planning and implementation of training schemes and development of transferable competencies and training materials. b) Mapping patient pathways and flow and c) Production of key documentation and Standard Operating Procedures for the delivery of the service.

**Results:**

*Phase 2 (Implementation and testing of the intervention):* During the initial 12 months of implementation, remedial action was taken to address identified deficiencies regarding patient referral to the pathway, compliance with treatment protocol, patient follow up, selection challenges from the use of PESI in cancer patients and challenges regarding the “first-pass” identification of i-PE.

**Conclusion:**

We have developed and piloted a complex intervention to manage cancer patients with incidental PE in an outpatient setting. Adherence to evidence- based care, improvement of communication between professionals and patients, and improved quality of data is demonstrated.

## Background

The Venous Thromboembolism (VTE) syndrome comprises deep vein thrombosis (DVT) and pulmonary embolism (PE) [1] and is particularly prevalent amongst cancer patients [2].

It is recognized that thrombosis and cancer are linked by multiple physiopathological mechanisms and that tumour biology and coagulation processes are integrally connected [3]. Therapeutic interventions including surgery and chemotherapy further increase the risk for thrombosis. Despite this being common knowledge the true prevalence of VTE is underestimated as clinical presentation may be truly asymptomatic and/or misattributed as well as the more recognised symptoms and signs of thrombosis [4,5].

The recent improvement in imaging technology, through the introduction of multi-slice spiral computed tomography (CT) scans, and the more frequent whole body imaging of patients with cancer due to the expansion in treatment options and trials, has led to an increasing incidence of incidental diagnosis of DVT or PE [6]. According to the official statement of the subcommittee on Haemostasis and Malignancy of the SCC of the International Society on Thrombosis and Haemostasis, incidental VTE in oncology patients is defined as “VTE identified in scans ordered primarily for staging or restaging of malignancy” and the terms “incidental” or “unsuspected” are recommended against “asymptomatic” [7]. Patients with cancer and incidentally diagnosed PE (i-PE) may be found to have symptoms on closer review, or may genuinely have no PE related symptoms [8,9]; however, the detrimental impact on survival is the same as for PE diagnosed following clinical suspicion [10]. Therefore, the standard of care remains to treat all cancer patients with a PE (or a DVT) irrespective of the manner of diagnosis. Most patients found to have a PE, in the UK, spend 5–7 days in hospital. The aim of admissions is to avoid potential complications such as death, progressive right ventricular failure and major bleeding while the patient is being established on anticoagulants, even though only 4.5% will develop serious complications during this time [11].

This ongoing practice may involve a significant number of inappropriate admissions and, for cancer patients, inappropriate anticoagulant management. The aim of this project therefore, was to develop an evidence based and protocol driven complex intervention (the i-PE pathway) to manage cancer associated i-PE in an outpatient setting, where safe and appropriate. A Working Group was constituted to oversee and establish the intervention based on the UK MRC Framework for developing complex interventions [12].

In the current article we present the development and implementation of the intervention, structured in three phases: Phase 0 (theoretical underpinning), Phase 1 (modelling of the i-PE pathway - March 2009 to February 2010) and the pilot phase (Phase 2) which includes the initial period of implementation, testing and real-time refinement and optimisation (March 2010 to September 2011). The described pathway is currently fully operational in its presented iteration as part of Acute Oncology services in our centre.

## Methods

### Phase 0. Theoretical underpinning

Published literature relevant to out-patient management of cancer associated PE.

Unfortunately, although there are recommended protocols for the management of cancer associated thrombosis, including outpatient management of DVT, there is very little guidance from existing literature to underpin outpatient management of PE (incidental or otherwise). A meta-analysis of trials of outpatient management of PE [13] included only two small retrospective series with cancer patients. The cancer patients were a ‘subgroup’ of PE patients managed as outpatients [14,15] but with no clear selection criteria. A further report on a larger cohort of 473 patients with acute PE showed that 55% were treated as outpatients. The decision not to admit was based on the emergency department doctor’s clinical judgment. Reasons to admit were “severe comorbidities” (46%) or hypoxia (22%) [11]. No validated risk assessment tool in this and most other studies was used prospectively to quantify short term risk of death, and in almost all, coexisting active cancer is assessed as necessitating admission.

#### Evidence on risk assessment tools

**Figure 1:**
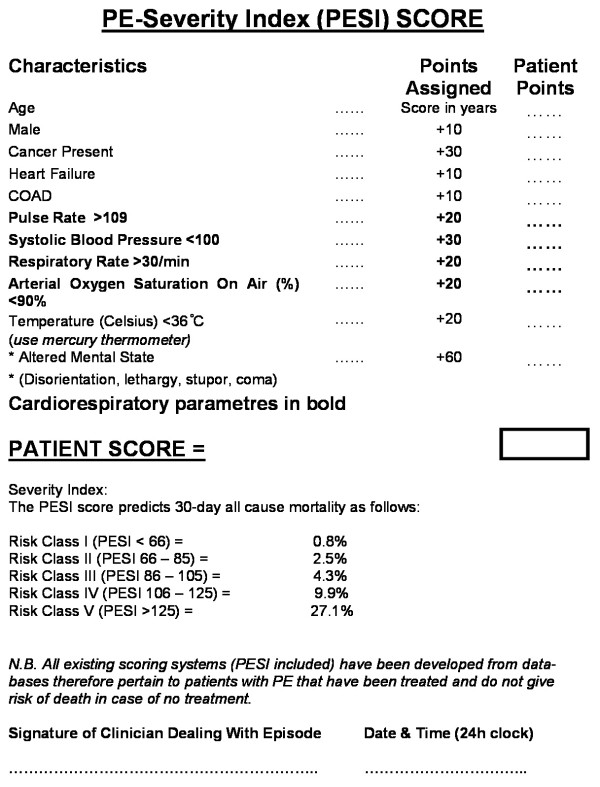
The Pulmonary Embolism Severity Index (PESI) scoring tool – modified layout for patient scoring as used in our pathway.

From the existing risk assessment tools the most robust seemed to be the Pulmonary Embolism Severity Index (Figure 1) [16]. This tool has 11 predictors scored from +10 to +60 identifying five risk classes (I–V). All indices are clinically generated and it is easy to administer. It has been validated prospectively [17] found to be superior to the GENEVA scoring system and it has low inter assessor variability [18].

A recent randomised controlled (RCT) trial in non-cancer patients with PE [19] which used this tool showed no difference in outcomes for those with low risk (score < 86) when managed as outpatients. Almost all cancer patients however have a PESI score of more than 100.

Two risk assessment tools specifically developed for cancer patients have only very recently been proposed and were not available at the time of the design of the intervention. POMPE-C [20] and RIETE [21] both predict mortality in patients with cancer and acute PE whilst POMPE-C has been also compared with PESI demonstrating higher prognostic accuracy in patients with active cancer [20]. These scores may become useful tools for prognostication of cancer patients with i-PE and our group will aim to assess these scores in this setting.

#### Guidance for treatment of established VTE/PE in cancer patients

In the absence of contemporaneous guidance from the National Institute for Health and Clinical Excellence (NICE) establishing standards of care, data from retrospective local audits were assessed against the recommendations of ASCO 2007 [22]. Current recommendations from all relevant bodies are similar and rely heavily on a published RCT [23].

The ASCO Recommendations for the management of established VTE in cancer patients can be summarised as follows: a) Low molecular weight heparin (LMWH) is the preferred approach for the initial 5 to 10 days, b) LMWH given for at least 6 months is also the preferred approach for long-term anticoagulant therapy. Vitamin K antagonists with a targeted INR of 2 to 3 are acceptable, c) indefinite anticoagulant therapy should be considered for selected patients with active cancer, such as those with metastatic disease or those receiving chemotherapy d) vena cava filters can be used in the presence of contraidications for anticoagulation or recurrent VTE while on adequate anticoagulation treatment. e) Anticoagulation should be avoided in the presence of active intracranial bleeding, recent surgery, pre-existing bleeding diathesis such as thrombocytopenia (platelet count < 50,000/μL) or coagulopathy and f) for elderly patients, anticoagulation is recommended for established VTE as described for other patients with cancer.

#### Audit findings and clinical activity data in relation to contemporaneous evidence

i) Audit

The predicted annual caseload of i-PE for a trust of the size of Hull and East Yorkshire Hospitals NHS Trust (HEY) is between 50–55 patients. A retrospective audit (spanning the period from August 2006 to January 2008–18 months) undertaken through the radiology department reporting database of our cancer centre (Hull and East Yorkshire Hospitals NHS Trust) identified 72 cases of incidental VTE (including visceral thrombosis) of which 58 were i-PE [24]. We estimated that this caseload represented 70-75% of the incidence over this period. Thirteen of these patients were already inpatients. Admission rate for outpatient diagnoses was 33%. We observed that the management of these patients was poorly coordinated (as indicated e.g. by long times from CT scan to treatment initiation) and was not uniform. Only in 34 patients (58%) management was assuredly consistent with ASCO recommendations.

ii) Data from patient registry

To establish a better understanding of the admission practice for i-PE patients across HEY, immediately prior the roll-out of the new pathway, a review of the clinical activity of the preceding 12 months was undertaken. Data was recovered from the Trust’s registry database for the period spanning from April 2009 to March 2010. All patients coded for PE were retrieved (345 patients) and the patients with the diagnosis of i-PE were analysed. 28 patients with cancer and i-PE diagnosed as outpatients had a registered admission with a mean hospital stay of 5.7 days (range: 0–12)

### Phase 1. Modelling phase

This phase aimed to clarify key elements (Table 1) for the intervention and their interactions and make the necessary adaptations for the pilot site. During this phase the appropriate personnel to be involved in the project multi-disciplinary management group were identified and consulted.

**Table 1:** Phase 1 modelling

**Elements**	**Implementation**
**a) identification of training needs and requirements/implementation of plans to meet these needs**	i) Nurse Specialists: Formalised training module (competency)
	ii) Radiographers: Case-review training material
**b) mapping of patient pathways and flow**	Flow charts
**c) production of key documentation and Standard Operating Procedures (SOP)**	i) scoring tool
	ii) questionnaires
	iii) prescription of medication
	iv) follow-up documentation: toxicity, complications, outcomes
	v) SOP/flow charts.

Key elements of the intervention.

#### Identification of training needs and implementation of training plans

It was recognised that the implementation of the pathway would need to be based on specific competencies of permanent non-medical staff including clinical nurse specialists (CNSs) and CT radiographers.

i) Clinical Nurse Specialists (CNSs)

A new competency for CNSs for the management of i-PE was introduced and ratified by the Trust Nursing and Midwifery Training and Education Forum. In the absence of existing literature or training ‘package’, key training needs taking in to consideration the lack of pre-existing competency, were laid out in a consultation document by AM and JP and further input was obtained from the University of Hull school of nursing. A comprehensive training schedule was produced that required the nurse practitioner to demonstrate knowledge and understanding through observation and assessment by an identified supervisor. Amongst the elements of the module are the understanding and implementation of the risk scoring tool; key cardio respiratory indices to be assessed; familiarity with the prescription medication (Dalteparin) and the recommended schedule (which includes a 25% dose reduction after week 4). Key knowledge such as an understanding of the adverse effects of Low Molecular Weight Heparin (commonly bleeding risk but also including the Heparin-Induced Thrombocytopenia) was also incorporated. Key categories of patients that would need immediate clinical referral out with the pathway were also identified (e.g. patient with GI bleed or brain metastases). Once these competencies were finalised a training module was developed which was mapped to the Hull & East Yorkshire Hospitals NHS Trust Systematic Training and Education Programme Level 2 (STEP 2) [25] (Additional files 1 and 2 show the supervisor and the practitioner packs as mapped to the STEP 2). These CNSs for the purposes of the manuscript are called ‘assessors’. Permission to use the PESI score [16] within the context of this service was obtained from Prof. Drahomir Aujesky (personal communication).

ii) Radiographers

The competency requirements of CT radiographers have been developed according to the document “The NHS Improvement Plan Putting People at the Heart of Public Services” [26]. In this document, incidental findings are described as “findings that are unrelated to the clinical indication for the imaging examination performed” and include a specific category of “emergency findings” defined as pathologies which alter patient management before their next outpatient appointment. Examples of emergency findings are pulmonary emboli, abdominal obstruction, destructive bone lesions, pneumothorax and brain metastases. In specific relevance to the incidental PE management pathway regular teaching is implemented to enhance identification of PE at this ‘first-pass’ opportunity of the patient in the department. Case review training material, such as a DVD of typical cases of PE distribution, has been produced to enhance learning.

#### Mapping of patient pathways and flow

Establishment of the new pathways was supported from within existing resource in HEY. Firstly a working group (WG) with operational responsibility was formed. The WG remit was to establish an evidence-based, protocol-driven complex intervention following UK MRC guidance and implement it for patients diagnosed with an i-PE. Continuing oversight of the project was provided by (MJ) who had previous expertise in the development of a complex intervention using this guidance. This WG met monthly and was attended by acute oncologists (at least one of LO’T, GB, AM), radiologist (GA), radiographer (AS), assessors (at least one of JP, ME, JH or SD), data manager and representative from the community based DVT service. A project specific database was written (GB) and a data manager (KM or VW) within radiology was tasked to collect and collate the data (initials correspond to contributors mentioned in the authors list and the acknowledgements list).

**Figure 2:**
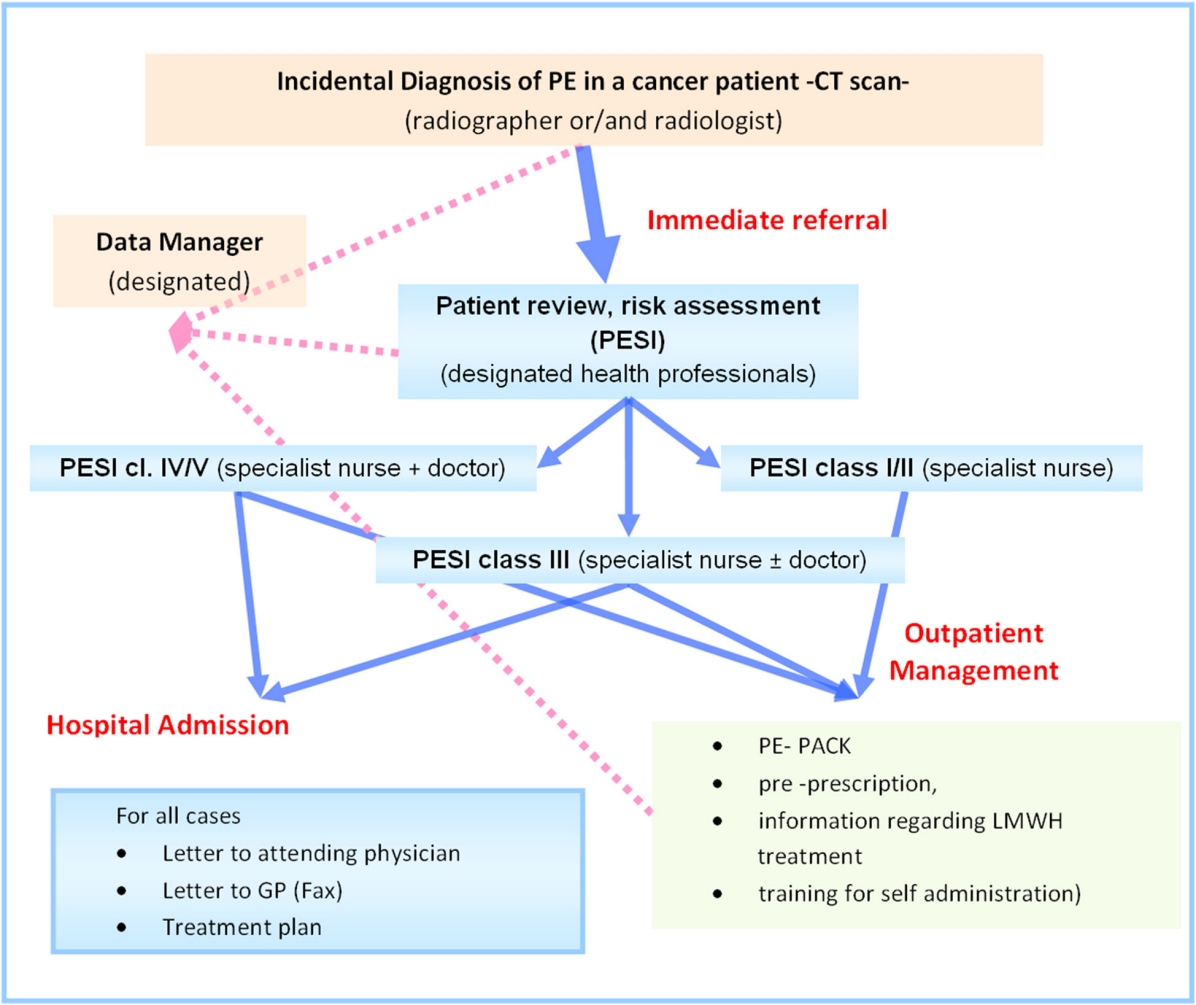
**Diagram of the i-PE pathway.** Patient flows in blue arrows. Data flow in dashed pink arrows.

The WG formulated the complex intervention that included the mapping of all pathways that lead from radiology to the end practitioner (assessor) who was to initiate management of the patient. The practitioner would assess the patient, administer appropriate questionnaire(s), initiate the treatment, refer the patient into the appropriate clinic for follow up, or arrange medical review/admission, inform community/GP practice and send information to the data manager for data recording (Figure 2).

#### Production of key documentation and standard operating procedures

The patient documentation used by the Chemotherapy Nurse Specialist Team (assessors) when assessing a newly referred patient includes a demographic sheet with observations performance status and weight and space for communicating the outcome of the assessment, a history checklist to determine patient history and a symptom questionnaire for the patient to complete (Additional file 3 shows the data-sheet and patient symptom questionnaire used).

A Pulmonary Embolism Severity Index (PESI) score form is also completed (Figure 1). Other documentation used includes a data sheet, Dalteparin prescription and a GP letter.

#### Follow –up and data collection

Initial data are collected during patient assessment by the assessors and entered onto the data base by the Data manager. Further data collection was planned to happen at opportune but not predefined intervals during the proposed period of LMWH treatment, to include early (day 0 and 7) platelet counts, capturing dose ‘step down’ at week 4, LMWH discontinuation, schedule-dose alteration or agent alteration (e.g. warfarinization), complications (bleeding, painful abdominal wall bruising) and quality of life concerns.

#### Dissemination/education

The Clinical Practice Development Committee reviewed the pathway (December 2009). Medical staff and allied professionals were informed of the complex intervention through a series of internal meetings prior to the project commencement in March 2010 and regular updates were communicated through existing specialist forums (radiology, nursing and oncology service meetings) when changes to the intervention were implemented.

### Summary iteration of resultant complex intervention entitled “The i-PE outpatient pathway”

The i-PE pathway resulting from the theoretical underpinning and modelling phase is summarised:

i) From Scanner to Assessor

As per the pathway, an incidental PE can be identified at two stages:

a) ‘First Pass’: By the scanning radiographer, who then brings it to the attention of the radiologist. Patient enters pathway on day of scan.

a) ‘Reporting Stage’: By the reporting radiologist. Patient enters pathway at the time of formal review of the CT, usually a few days after scan.

In practice all out patient scans are reviewed by radiographer prior to the patient leaving the department aiming to identify any emergency findings. When a PE is identified by the radiographers this is brought to the attention of the CT supervising radiologist and if the finding is verified, action is taken according to the pathway recommendations to ensure correct management of the patient. A total of four discrete notification/referral pathways were established to cover working hours and out-of-hours identification of i-PE during scanning (Radiographer) or during reporting (Radiologist) respectively (flow charts depicting these pathways can be found in Additional file 4).

ii) From Practitioner to Community (or admission)

The telephone referral from the radiologist is received by the assessors and the patient is seen within 24 hours of the referral being received. The patient is assessed, PESI score recorded and all relevant documentation completed.

For patients that are risked assessed and presumed to have a high potential risk of 30-day mortality [PESI score (> 125)] [16] or patients with abnormal indices on the cardio-respiratory criteria of the PESI scoring system and/or new symptoms an acute oncology medical review is undertaken and admission considered.

When the patient is deemed eligible for out-patient care, he/she or his/her carer is trained how to inject low molecular weight heparin (LMWH) and the patient is provided with the first 4 weeks of treatment.

This process takes approximately one hour. All patients undergo blood sampling for a full blood count and a biochemical profile including renal function tests at baseline and repeated seven days after treatment is initiated. Prior to leaving the hospital the patient is given a follow up appointment with their treating clinician. A letter, containing a treatment plan, is faxed to the patients’ general practitioner (GP) informing them of the i-PE and the treatment initiated. The hospital provides the patient with enough LMWH, (Dalteparin) at a dose of 200 iu/kg to last them for 4 weeks. After 4 weeks the dose is stepped down to 150 iu/kg as per product recommendations and the patient continues on this dose for the next 5 months in accordance with current guidance. It is expected that after the first 4 weeks, the patients’ GP will initiate the step down dose and provide future injections until treatment is discontinued or the patient is warfarinised.

The pathway also considers the i-PE patient who, according to the diagnosing radiographer’s opinion, is unwell and should be seen immediately. This is specifically relevant for scanning lists that are out of hours or on weekends as the intervention is available only within working hours-. Referral into the acute oncology referral pathway is recommended and incorporated in the pathways.

## Results and discussion

### Phase 2: Pilot. Real time re-modelling, refinement and optimization of the complex intervention

From March 2010 to September 2011, 82 patients had been managed using the i-PE pathway. Data on this group have been presented in abstract form [27] and demonstrate marked improvements in admission rates and guideline adherence.

In the regular WG meetings, ongoing modelling of the pathway was possible in response to problems and deviations arising in practice, resulting in real-time remedial action.

#### Patients managed independently of the i-PE pathway

HEY operates two CT diagnostic units in its two major sites (Hull Royal Infirmary-HRI and Castle Hill Hospital-CHH) with the Accident and Emergency (A&E) and Oncology departments on different sites, six miles apart. In addition, since the incidence patterns of i-PE are unpredictable the assessors cannot reserve clinic time. Pathway patients are therefore seen alongside the usual daily workload. In practical terms immediate assessor availability is not universally (100%) possible.

This operational structure was identified by the WG as the main underlying reason behind the cases where patients failed to be referred properly and be managed within the pathway. In total, 6 such patients were identified. Three patients were admitted directly via A&E and the assessors were not informed. Two patients were already receiving LMWH, were managed by their treating clinician and were not referred. One patient was still undergoing diagnostic investigations for cancer and was managed by the diagnosing clinician.

##### Remedial action

In order to improve the co-ordination of patients diagnosed with i-PE at the time of scanning, the radiology pathways were adjusted to give better direction to the radiologist and reinforce the i-PE pathway referral algorithms, with care to cover circumstances when the assessors are not immediately available or the distance between the HRI CT site and Oncology in CHH (where the nurse practitioners are based) is a factor for immediate review by the team. These adjustments enabled patients who were asymptomatic to be allowed home and invited back for assessment, rather than being admitted. For patients who are symptomatic, especially when diagnosed out of hours, the pathway was modified to explicitly instruct radiologists to arrange the review of the patient by the Acute Oncology out of hours service in the Oncology unit and not the standard A&E service so that cases are rapidly and centrally assessed and managed according to the set guidelines. All such cases are also reviewed by the recognised assessors at the earliest convenience and data is captured as per the pathway.

#### Failures of compliance with dosing protocol

After the first three months of the pathway being implemented the patients referred onto the pathway were contacted to ensure that their dose of Dalteparin had been stepped down. It was discovered that GPs were not stepping down the dose of heparin after the first 4 weeks as instructed. On an interim survey of the first 40 patients managed with the pathway, the rate of dose de-escalation at four weeks was 40% (n = 14, no data for 5 patients).

##### Remedial action

This was addressed by adjusting the wording in the GP letter to highlight the date of projected step down. Furthermore patients were educated to expect/request the step down at 4 weeks.

#### Unreliable capture of follow up data

It became apparent that reliable toxicity data or information whether the patients continued on LMWH were difficult to obtain from the source notes or the hospital patient information system.

##### Remedial action

The initial documentation was modified to gain consent from patients for the assessor to contact them by telephone at 3 months and 6 months.

#### Selection challenges from the use of the PESI tool

Even before the intervention commenced the WG recognised that the use of a generic pulmonary embolism scoring system in a specific subgroup of patients (i.e. cancer patients with i-PE) could pose particular selection challenges, the nature of which however was difficult to predict. Prospective collection therefore of indices that are known to have predictive properties for cancer was included (e.g. performance status, stage of disease). The interim analysis of the data (first 82 patients) revealed a significant overestimation of mortality risk by the PESI in this subgroup of patients [27].

##### Remedial action

Modification of the risk assessment tool was done to a) add a ‘new symptoms’ index that the emerging data seemed to suggest was a stronger predictor of worse outcome and b) allow greater discharge latitude to the assessors based on the audit data of the first 82 patients.

#### ‘First pass’ Identification of i-PE

Given that identification of i-PE by radiographers was a major component of the service improvement and reinforced within the training competency a post-implementation prospective audit was undertaken.

In our recently published analysis [27] we found that i-PE was identified at the time of scanning by the radiographers in 56 (71%) of 79 patients (Table 2). Among the 23 cases missed by radiographers, n = 17 (74%) were segmental or sub-segmental and n = 7 (26%) were central or lobar.

**Table 2:** i-PE identification rates for radiographers, per anatomical site

	Number of patients*	**Identified by radiographer (%)**
**All**	79	
**Central PE**	20	15 (75)
**Lobar PE**	9	7 (78)
**Segmental PE**	41	29 (70)
**Subsegmental**	9	5 (56)

* Includes 79 of 82 patients presented in ISTH 2012 [27]. Information regarding identification of PE by the radiographer was missing in 3 patients (4%).

The reasons for this ‘miss rate’ were found to include the following: a) In the busy scanning environment radiographers can be distracted by telephone calls, control room queries, and pressure to scan the next patient, therefore omitting the review or not reviewing with sufficient time/scrutiny. b) Inexperienced staff may miss PE due to lack of experience/education.

Also noted is that the i-PE pathway received three inappropriate referrals from radiology at the same period: a) one did not have a PE, b) one was referred for further investigations and c) one patient was a suspected but not verified DVT and not PE.

##### Remedial action

Processes such as ongoing education of radiographers, improved and more frequent feedback and training in a systematic method of reviewing the scans is being implemented. Detection rates will be audited again in a future cohort. To reduce false positives a formal CT report of the PE to be dictated and made available for review by the assessor prior to patient assessment.

### Observations on Phase 2 of the intervention

This pathway has standardised practice for the care and management of this particular patient group. Early repeat audit data indicate an important increase in the number of patients safely managed at home rather than in hospital allowing not only cost savings for the institution, but, more importantly, patient benefit in prevention of unnecessary hospital admission. The implementation of the pathway has improved communication between healthcare professionals and hence the quality of data acquisition Confidence gained from working in this setting has also motivated the staff to seek training in further skills. All the assessors are including the prescription of LMWH in their training as supplementary prescribers while continued education of radiographers to improve ‘first pass’ identification of PE is ongoing and has resulted in the production of a training DVD. Preliminary key findings have been that 71% of patients were diagnosed by radiographer on the same day of scanning and the majority of patients 57% are reviewed the same day as the CT scan (range 0–21) [27]. Adherence to guidelines has also improved markedly. We recorded adherence to ASCO 2007 guidelines of 98% [27] in the group of patients managed prospectively on this pathway compared to 58% from our retrospective audit.

The development and implementation process has focussed on the specific issues relating to our NHS Hospital Trust nevertheless our experience has led to a deeper understanding of the ‘modular’ transferable elements for adaptation to and implementation in other NHS hospital environments.

Resource utilization is also likely to be improved and possible cost-savings realised; however this work did not have health economics planned and although simple retrospective calculations based either on the cost of occupying a bed in the Trust or on the national tariff for uncomplicated PE may suggest significant cost savings, one has to be critical of ad hoc methodology employed to these data. This service model would need to be exposed to a proper resource utilization analysis within a prospective trial-setting designed to establish the true value/resource implication of a restructured service that embraces this intervention. Similarly although we believe that the avoidance of admission should enhance the patient experience we do not have any comparative evidence to show this. For example it is possible that for some patients at least the anxiety of being discharged with a ‘clot in the lungs’ may be significant and an admission for them may seem preferable. So prospectively well designed qualitative studies to capture these elements between the two service models would be needed.

## Conclusions

We have developed and piloted a complex intervention for the outpatient management of i-PE in cancer patients, based on MRC UK Guidance. This complex intervention has core components which can be mapped and adapted for use in other NHS organizations. The pilot phase has demonstrated improved flow of patients through departments, improved communication between professionals and patients, and improved adherence to evidence based guidelines. Initial findings suggest that using this intervention most patients can be safely and effectively managed in an outpatient setting with improvement of quality of care.

## Abbreviations

ASCO: American Society Clinical Oncology; CHH: Castle Hill Hospital; DVT: Deep Vein Thrombosis; HEY: Hull and East Yorkshire; HRI: Hull Royal Infirmary; INR: International Normalised Ratio; i-PE: Incidental pulmonary embolism; LMWH: Low Molecular Weight Heparin; MRC: Medical Research Council; PESI: Pulmonary Embolism Severity Index; RCT: Randomised controlled trial; STEP: Systematic training and education for practice; WG: Working group.

## Competing interests

The authors declare that they have no competing interests.

## Authors’ contributions

JP - Designed and implemented training- Supervisor Assessor- Member WG group- Co-wrote edited, corrected and approved the manuscript. GB: Wrote the data-base and supervised data acquisition- Member WG group- Co-wrote, analysed data, co-wrote, edited, corrected and approved the manuscript, corresponding author. AS: Designed patient flows from radiology. Designing and implementing radiographer training. Member WG. Edited, co-wrote and approved the manuscript. MJ - WG-Expertise with MRC guidance for Complex interventions, co-wrote edited and approved manuscript. GA: Designed patient flows from radiology. Designing and implementing radiographer training. Member WG. Edited and approved the manuscript. LO’T: Member of WG. Designing acute outpatient oncology services. Edited and corrected the manuscript. AM: Service Design conception. Designed and implemented training- Supervisor-Trainer- Member WG- Co-wrote edited, corrected and approved the manuscript. All authors read and approved the final manuscript.

## Pre-publication history

The pre-publication history for this paper can be accessed here:

http://www.biomedcentral.com/1472-6963/13/235/prepub

## Supplementary Material

Additional file 1 **Supervisor (a) pack.** Hull & East Yorkshire Hospitals NHS Trust Systematic Training and Education Programme (STEP) Level 2. Click here for file

Additional file 2 **Clinical Nurse Specialist (b) pack.** Hull & East Yorkshire Hospitals NHS Trust Systematic Training and Education Programme (STEP) Level 2. Click here for file

Additional file 3 Demographics and management data-sheets, patient symptom questionnaire. Click here for file

Additional file 4 Notification/referral pathways. Click here for file
