# Supplementary material for: Developing a complex intervention for the outpatient management of incidentally diagnosed pulmonary embolism in cancer patients
Source: BMC Health Serv Res. 2013 Jun 27;13:235. doi: 10.1186/1472-6963-13-235 (PMC3718646; doi:10.1186/1472-6963-13-235)
Supplement: Additional file 1 — Supervisor (a) pack. Hull & East Yorkshire Hospitals NHS Trust Systematic Training and Education Programme (STEP) Level 2. [file 1472-6963-13-235-S1.pdf]

Hull and East Yorkshire Hospitals NHS Trust

THE QUEEN'S CENTRE FOR ONCOLOGY AND HAEMATOLOGY

The Management of Cancer Patients with Incidental Pulmonary Embolism

Supervisors Pack

Version:	2.0
Date Published:	March 2012
Review Date:	November 2012
Authors:	Dr A. Maraveyas, Consultant Medical Oncologist
	June Palmer, Chemotherapy Nurse Specialist

Step 2 Practice	Core Competencies Element<br>Incidental Pulmonary Embolism and Venous Thromboembolism

Overview of Element

Many common clinical problems in medicine and general practice relate to arterial and venous thrombosis. Venous Thromboembolism (VTE) is a frequent problem in patients with cancer and represents a major clinical complication. Previously oncology patients were almost twice as likely to die of PE as those patients with benign disease and about 60% of those deaths occurred prematurely. Therapeutic treatments including chemotherapy and surgery further increase the risk for thrombosis. Unfortunately there are few standardised protocols for the management of oncology patients who develop VTE and care can often be fragmented. Problems identified include poor communication between health care professionals and patients.

An audit was undertaken July-October 2008 to assess the incidence and management of incidental VTE in cancer patients in Hull and East Yorkshire Hospitals NHS Trust. It was apparent from the audit that there was no single standard of care, or any documentation of assessment, treatment outcome and complications. Moreover, the literature suggested that the majority of these patients could probably be managed as outpatients with significant cost saving implications.
A pathway and documentation has now been established and the plan is to manage patients based on this pathway and re audit the findings over the next year.

The aim is to provide a high quality, flexible and equitable service to all oncology/ haematology patients within the Humber and Yorkshire Cancer Coast Network who require management of incidental VTE.

The practitioner undertaking patient assessment must be:

- a band 7 working within the Chemotherapy Nurse Specialist Team
- have been assessed as competent following a period of observed practice.
- have undertaken training on the PE-Severity Index Score.

Training and assessment will be undertaken by Dr Maraveyas, Consultant Medical Oncologist.

Practitioner workbook can be found on pages 6 – 15 of the Practitioner Pack.

Sign off sheet can be found on page 8 of the Supervisor pack.

Copyright © Hull and East Yorkshire Hospitals NHS Trust 2012

Step 2 Practice	Core Competencies Element<br>Incidental Pulmonary Embolism and Venous Thromboembolism

Outcome Statement

In order to achieve safe assessment and management of patients found to have incidental PE/VTE, the practitioner will have knowledge and understanding of:

- Aetiology of coagulation and thrombocytopenia
- Heparin induced thrombocytopenia (HIT)
- Normal/abnormal blood results including clotting/D-dimer results
- Pulmonary Embolism Severity Index (PESI) scoring classification

Source Documents:-

British National Formulary: 2007, British National Formulary 54

http://www.bnf.org/bnf/bnf/54/59028.htm#this

Department of Health: 2000 The NHS plan, http://www.dh.gov.uk

Department of Health: 2006, Our Health, Our Care, Our Say.

http://www.dh.gov.uk/en/Policyandguidance/Organisationpolicy/Modernisation/Ourhealthourcareoursay/index.htm

The Case for Commissioning an Incidental PE Pathway – A. Maraveyas, G. Avery & M.Johnson.

Thrombosis, (2006). PE and the benefits of LMWH. Vol 10, 4.

HEY Policy No. CP 016 Consent to Examination or Treatment.

HEY Policy No. CP 26 Drug Policy.

http://www.labtestonline.org.uk

Martini F.H & Nath J.L, (2009) Fundamentals of Anatomy & Physiology, 8th Edition, Pearson International, London.

Higgins C, (2007) Understanding Laboratory Investigations for Nurses and Health Professionals, 2nd Edition, Blackwell Publishing, Oxford.

Copyright © Hull and East Yorkshire Hospitals NHS Trust 2012

Step 2 Practice	Core Competencies Element<br>Incidental Pulmonary Embolism and Venous Thromboembolism

Assessment Methodology: - **O = Observation**
Q = Questioning
Ukn = Underpinning Knowledge/portfolio evidence

Outcome:

- For Practitioners to have the knowledge and level of competence to provide high quality anticoagulation management for Oncology/Haematology patients who develop incidental PE/VTE.

Clinical Competency	Evidence
**1.0**<br>Practitioner can demonstrate knowledge and understanding of coagulation.	The coagulation cascade can be thought of as having two branches: the extrinsic pathway and the intrinsic pathway. Each of these pathways utilizes different coagulation factors, proteins that are carried in an inactive form in the blood. These factors are activated in sequence down one pathway or another and come together to complete the clotting process, the common pathway.<br><br>The extrinsic pathway begins with the release of Factor III, or Tissue Factor (TF), by damaged endothelial cells or peripheral tissue. The greater the damage, the more tissue factor is released and the faster the clotting occurs.<br>Tissue Factor then combines with Factor XII to form a complex enzyme capable of activating Factor X.<br>The activation of Factor X is the first step in the common pathway.<br><br>The intrinsic pathway begins with the activation of pro enzymes (usually Factor XII) exposed to collagen fibres at the injury site. This pathway proceeds with the assistance of PF-3, a platelet factor released by aggregating platelets. Platelets also release a number of other factors that accelerate the reaction of the intrinsic pathway. After a series of linked reactions, Factors VII and IX combine to form an enzyme complex capable of activating Factor X.<br><br>The common pathway begins when enzymes from either pathway activate Factor X, forming the enzyme Prothrombinase. This converts the proenzyme Prothrombin into the enzyme Thrombin. Thrombin then completes the clotting process by converting Fibrinogen into insoluble strands of Fibrin (a blood clot is formed).<br>Work book evidence.

Copyright © Hull and East Yorkshire Hospitals NHS Trust 2012

Step 2 Practice	Core Competencies Element<br>Incidental Pulmonary Embolism and Venous Thromboembolism

**1.1**<br>Practitioner can demonstrate knowledge and understanding of Heparin Induced Thrombocytopenia and Thrombosis (HITT).	Heparin Induced Thrombocytopenia and Thrombosis (HIT) is a drug induced immune mediated syndrome characterized by thrombocytopenia and thrombotic events that may be life or limb threatening.<br><br>HIT can occur in approximately 5% of patients receiving unfractionated heparin and in less than 1% of patients receiving low molecular weight heparin.<br><br>In HIT the immune system forms antibodies against heparin when it is bound to Platelet Factor 4. The resulting antibody response activates platelets and the formation of platelet micro particles.<br>This complex event can cause both the thrombocytopenia and thrombosis seen in HIT.<br><br>Despite thrombocytopenia which can be severe, bleeding complications are rare and patients with HIT are actually at higher risk of thrombosis. HIT is associated with a high risk of new thrombosis or extension of existing thrombosis.<br><br>HIT can occur anywhere between 5 to 10 days after initiation of treatment.<br><br>If HIT is suspected, heparin therapy should be discontinued immediately, Warfarin should never be used and the patient anticoagulated with a direct thrombin inhibitor or Fondaparinux. Patients with HIT should never be left without anticoagulation.<br><br>Work book evidence.
**1.2**<br>Practitioner can demonstrate knowledge and understanding of normal/abnormal coagulation screen/D-dimer results.	Prothrombin Time (PT), Activated Partial Thromboplastin Time (APTT) and Thrombin Time (TT) all test the ability of the blood to generate Fibrin by the blood clotting cascade.<br>They all measure the time taken for a sample of the patient’s blood to form a Fibrin clot in a test tube, after a reagent had been added which initiates the clotting cascade.<br>An increase in any of these tests indicates a deficiency of one or more clotting factors.<br><br>D-dimer test is used to help diagnose or exclude thrombotic diseases and conditions, usually in conjunction with a pre diagnosis risk of thrombosis assessment.<br>A positive D-dimer indicates the presence of an abnormally high level of cross linked fibrin degradation products in the body. It tells the doctor that there has been significant clot formation and breakdown in the body, but, it does not identify the location of the clot or the cause.<br>An elevated D-dimer may be due to Venous Thromboembolism (VTE) or Disseminated Intravascular Coagulation (DIC).<br><br>A normal D-dimer test means it is unlikely that the patient has an acute

Copyright © Hull and East Yorkshire Hospitals NHS Trust 2012

	blood clot or disease causing abnormal clot formation and breakdown (negative predictive value is high).<br><br>D-dimer is recommended as an “additional” test and should not be the only test used to diagnose a disease or condition.<br><br>There is evidence that in patients diagnosed with a PE/VTE, a persistently elevated D-dimer result taken 4 weeks after the period of conventional anticoagulation has concluded can indicate a high risk of relapse. There is also some evidence linking the levels of D-dimer to pulmonary embolism severity.
**1.3**<br>Practitioner can demonstrate knowledge and understanding of the Pulmonary Embolism Severity Index (PESI) scoring classification.<br>See appendix I.	The Pulmonary Embolism Severity Index (PESI) is a risk stratification tool for patients diagnosed with Pulmonary Embolism.<br><br>PESI can help to identify patients with very low adverse event rates during the initial stages of acute Pulmonary Embolism and assist in selecting patients for treatment in the outpatient setting, but, also to identify patients that may need ‘escalation’ to thrombolysis and HDU earlier.
**1.4**<br>Practitioner can demonstrate correct procedure for completing Pulmonary Embolism Severity Index (PESI) score and act accordingly.	Observation of practice.<br><br>a) PESI score <105 – Patient managed as outpatient.<br>b) PESI score >105 – Admission necessary, seek clinical advice if necessary.<br>c) PESI score >120 – Admit and inform senior clinician responsible for the patient.
**1.5**<br>Practitioner can demonstrate the correct patient assessment and clarify the purpose of the assessment.	Observation of practice.<br><br>Practitioner completes relevant assessment tools:<br>▪ Incidental Pulmonary Embolism Symptom Chart.<br>▪ PESI score.<br>▪ Incidental Pulmonary Embolism Management Data Sheet.<br>▪ History Checklist for Nurse Led Management of Cancer Patients with Incidental PE/VTE.

Copyright © Hull and East Yorkshire Hospitals NHS Trust 2012

Step 2 Practice	Core Competencies Element<br>Incidental Pulmonary Embolism and Venous Thromboembolism

**1.6**<br>Practitioner can discuss with the patient the correct procedure for taking prescribed Fragmin medication, confirms safe storage of Fragmin and obtains verbal consent for treatment.	Observation of practice.<br><br>Patient provided with Fragmin patient pack containing:<br>▪ Patient Information Booklet,<br>▪ Instructional DVD and leaflet on How to Administer Fragmin Injection using a pre filled syringe.<br>▪ Emergency contact number.<br>▪ Sharps bin.<br><br>Fragmin prescribed by clinician and 4 weeks supply dispensed by pharmacy.
**1.7**<br>Practitioner can demonstrate safe procedure for administering subcutaneous injection and confirm patient self management of injection/referral to district nurse.	Observation of practice.<br><br>Practitioner administers first injection and demonstrates to patient the correct procedure for administering sub cutaneous injection.<br><br>Practitioner clarifies patient self management of injection of referral to District Nurse.<br><br>Practitioner completes referral to District Nurse if necessary.
**1.8**<br>Practitioner can demonstrate the correct completion of appropriate documentation and ensures patient held records are completed.<br><br>Documents include:<br>• Patient demographics.<br>• Incidental pulmonary embolism symptom chart.<br>• Pulmonary embolism severity index score (PESI).<br>• Incidental pulmonary embolism management data sheet.<br>• History checklist.<br>• Dalteparin for outpatient treatment of pulmonary embolism – GP letter.	Observation of practice.<br><br>Completes patient demographics and assessment tools.<br>Documents outcome and intervention.

Copyright © Hull and East Yorkshire Hospitals NHS Trust 2012

Step 2 Practice	Core Competencies Element<br>Incidental Pulmonary Embolism and Venous Thromboembolism

**1.9**<br>Practitioner can describe the care and management of cancer patients with recurrent VTE.	Check “peak” anti factor Xa levels.<br>This should be taken about 4 hours after a LMWH dose is given, when it is expected that the concentration of LMWH in the blood is at its highest level.<br><br>For patients on Warfarin, switch to full dose LMWH.<br>For patients on the stepped down dose of LMWH, increase back to full dose.<br><br>Reassess in 5-7 days.<br><br>If on full dose of LMWH and anti Xa level is <1, adjust LMWH upwards by 25% and reassess symptoms and anti factor Xa levels in 5-7 days. If anti factor Xa level is >1 discuss with clinician.<br><br>If symptomatic improvement, continue full dose and resume usual follow up.<br><br>No improvement, continue full dose, but, consider twice daily dosing.<br><br>Workbook evidence

Copyright © Hull and East Yorkshire Hospitals NHS Trust 2012

Step 2 Practice	Core Competencies Element<br>Incidental Pulmonary Embolism and Venous Thromboembolism

Following a period of observed practice and on completion of Practitioner Workbook, practitioner can be signed off as competent to assess and manage patients diagnosed with incidental Pulmonary Embolism and Venous Thromboembolism.

Date:

Supervisor's comments:

Competency achieved ☐

Requires further training. ☐

If practitioner requires further training an action plan must be completed.

Signature of Practitioner……… ………………………………………….

Signature of Assessor…………………………………………………..

Copyright © Hull and East Yorkshire Hospitals NHS Trust 2012

Step 2 Practice	Core Competencies Element<br>Incidental Pulmonary Embolism and Venous Thromboembolism

Action Plan

Section	Action Plan	Timescale

Signature of Practitioner……… ………………………………………….Date………………..

Signature of Assessor…………………………………………………..Date………………..

Copyright © Hull and East Yorkshire Hospitals NHS Trust 2012

Appendix I

PE-Severity Index (PESI) SCORE

Characteristics		Points Assigned	Patient Points
Age	……	Score in years	……
Male	……	+10	……
Cancer Present	……	+30	……
Heart Failure	……	+10	……
**COAD**	**……**	**+10**	**……**
**Pulse Rate >109**	**……**	**+20**	**……**
**Systolic Blood Pressure <100**	**……**	**+30**	**……**
**Respiratory Rate >30/min**	**……**	**+20**	**……**
**Arterial Oxygen Saturation On Air (%) <90%**	**……**	**+20**	**……**
Temperature (Celsius) <36˚C<br>(*use mercury thermometer)*	……	+20	……
* Altered Mental State	……	+60	……

* (Disorientation, lethargy, stupor, coma)

Cardiorespiratory parametres in bold

PATIENT SCORE =

Severity Index:
The PESI score predicts 30-day all cause mortality as follows:

Risk Class I (PESI < 66) = 0.8%
Risk Class II (PESI 66 – 85) = 2.5%
Risk Class III (PESI 86 – 105) = 4.3%
Risk Class IV (PESI 106 – 125) = 9.9%
Risk Class V (PESI >125) = 27.1%

N.B. All existing scoring systems (PESI included) have been developed from data-bases therefore pertain to patients with PE that have been treated and do not give risk of death in case of no treatment.

Signature of Clinician Dealing With Episode **Date & Time (24h clock)**

……………………………………………………….. …………………………..

Copyright © Hull and East Yorkshire Hospitals NHS Trust 2012
