# Supplementary material for: Developing a complex intervention for the outpatient management of incidentally diagnosed pulmonary embolism in cancer patients
Source: BMC Health Serv Res. 2013 Jun 27;13:235. doi: 10.1186/1472-6963-13-235 (PMC3718646; doi:10.1186/1472-6963-13-235)
Supplement: Additional file 2 — Clinical Nurse Specialist (b) pack. Hull & East Yorkshire Hospitals NHS Trust Systematic Training and Education Programme (STEP) Level 2. [file 1472-6963-13-235-S2.pdf]

Hull and East Yorkshire Hospitals NHS Trust

THE QUEEN'S CENTRE FOR ONCOLOGY AND HAEMATOLOGY

The Management of Cancer Patients with Incidental Pulmonary Embolism

Practitioners Pack

Version: 2.0

Date Published: March 2011

Review Date: November 2012

Authors: Dr A. Maraveyas, Consultant Medical Oncologist

June Palmer, Chemotherapy Nurse Specialist

Step 2 Practice	Core Competencies<br>Incidental Pulmonary Embolism and Venous Thromboembolism

Practitioner Name	Supervisor Name
Date commenced	Date completed

Overview of Element

Many common clinical problems in medicine and general practice relate to arterial and venous thrombosis. Venous Thromboembolism (VTE) is a frequent problem in patients with cancer and represents a major clinical complication. Previously oncology patients were almost twice as likely to die of PE as those patients with benign disease and about 60% of those deaths occurred prematurely. Therapeutic treatments including chemotherapy and surgery further increase the risk for thrombosis. Unfortunately there are few standardised protocols for the management of oncology patients who develop VTE and care can often be fragmented. Problems identified include poor communication between health care professionals and patients.

An audit was undertaken July-October 2008 to assess the incidence and management of incidental VTE in cancer patients in Hull and East Yorkshire Hospitals NHS Trust. It was apparent from the audit that there was no single standard of care, or any documentation of assessment, treatment outcome and complications. Moreover, the literature suggested that the majority of these patients could probably be managed as outpatients with significant cost saving implications.

A pathway and documentation has now been established and the plan is to manage patients based on this pathway and re audit the findings over the next year.

The aim is to provide a high quality, flexible and equitable service to all oncology/ haematology patients within the Humber and Yorkshire Cancer Coast Network who require management of incidental VTE.

The practitioner undertaking patient assessment must:

- be a band 7 working within the Chemotherapy Nurse Specialist Team
- have been assessed as competent following a period of observed practice.
- have undertaken training on the PE-Severity Index Score.

Training and assessment will be undertaken by Dr Maraveyas, Consultant Medical Oncologist.

Practitioner workbook can be found on pages 6 – 15 of the Practitioner Pack.

Sign off sheet can be found on page 8 of the Supervisor pack.

Copyright © Hull and East Yorkshire Hospitals NHS Trust 2012

Outcome Statement

In order to achieve safe assessment and management of patients found to have incidental PE/VTE, the practitioner will have knowledge and understanding of:

- **Aetiology of coagulation and thrombocytopenia**
- **Heparin induced thrombocytopenia (HIT)**
- **Normal/abnormal blood results including clotting/D-dimer results**
- **Pulmonary Embolism Severity Index (PESI) scoring classification**

Source Documents:-

British National Formulary: 2007, British National Formulary 54

http://www.bnf.org/bnf/bnf/54/59028.htm#this

Department of Health: 2000 The NHS plan, http://www.dh.gov.uk

Department of Health: 2006, Our Health, Our Care, Our Say.

http://www.dh.gov.uk/en/Policyandguidance/Organisationpolicy/Modernisation/Ourhealthourcareoursay/index.htm

The Case for Commissioning an Incidental PE Pathway – A. Maraveyas, G. Avery & M. Johnson.

Thrombosis, (2006). PE and the benefits of LMWH. Vol 10, 4.

HEY Policy No. CP 016 Consent to Examination or Treatment.
HEY Policy No. CP 26 Drug Policy.

Copyright © Hull and East Yorkshire Hospitals NHS Trust 2012

For Registered Band 7 Practitioners.

Core / Specific	Number	Dimension	Second Gateway (Full Outline) Level	Indicator
Core	C1	Communication	4	a,b.c,d,e,f
Core	C2	Personal And People Development	4	a,b,c,d,e,f,g, h
Core	C3	Health, Safety And Security	3	a,b,c,d,e
Core	C4	Service Improvement	2	a,b,c,d,e,f
Core	C5	Quality	3	a,b,c,d,e,f,g
Core	C6	Equality And Diversity	3	a,b,c,d,e
Specific	HWB2	Assessment And Care Planning To Meet Health And Wellbeing Needs	4	a,b,c,d,e,f,g, h
Specific	HWB5	Provision of care to meet health and wellbeing needs	4	a,b,c,d,e,f,g
Specific	HWB7	Interventions And Treatments	4	a,b,c,d,e,f,g, h
Specific	IK1	Information Processing	1	a,b,c,d,e
Specific	G1	Learning & Development	3	a,b,c,d,e,f
Specific	G5	Service & Project Management	2	a,b,c,d,e,f,g

Copyright © Hull and East Yorkshire Hospitals NHS Trust 2012

Step 2 Practice	Core Competencies<br>Incidental Pulmonary Embolism and Venous Thromboembolism

Assessment Methodology: -

O = Observation
Q = Questioning
Ukn = Underpinning Knowledge/portfolio evidence

Outcome:

- For Practitioners to have the knowledge and level of competence to provide high quality anticoagulation management for Oncology/Haematology patients who develop incidental PE/VTE.

Clinical Competency	KSF Dimension	KSF Level	KSF Indicator
1.0<br>Practitioner can demonstrate knowledge and understanding of coagulation.	C2	4	a,b,c
1.1<br>Practitioner can demonstrate knowledge and understanding of Heparin Induced Thrombocytopenia (HIT).	C2	4	a,b,c
1.2<br>Practitioner can demonstrate knowledge and understanding of normal/abnormal coagulation screen/D-dimer results.	C2	4	a,b,c
1.3<br>Practitioner can demonstrate knowledge and understanding of the Pulmonary Embolism Severity Index (PESI) scoring classification.	C2	4	a,b,c
1.4<br>Practitioner can demonstrate correct procedure for completing Pulmonary Embolism Severity Index (PESI) score and act accordingly.	C2	4	a,b,c

Copyright © Hull and East Yorkshire Hospitals NHS Trust 2012

Step 2 Practice	Core Competencies<br>Incidental Pulmonary Embolism and Venous Thromboembolism

Clinical Competency	KSF Dimension	KSF Level	KSF Indicator
1.5<br>Practitioner can demonstrate the correct patient assessment and clarify the purpose of the assessment.	C1<br>HWB2<br>HWB5<br>IK1	4<br>4<br>4<br>1	a,b,c,d,e,f<br>a,b,c,d,e,f,g<br>a,b,c,d,g<br>a,b,d,e
1.6<br>Practitioner can discuss with the patient the correct procedure for taking prescribed Fragmin medication, confirms safe storage of Fragmin and obtains verbal consent for treatment.	C1<br>HWB7	4<br>4	a,b,c,d,e,f<br>a,b,c,d,g,h
1.7<br>Practitioner can demonstrate safe procedure for administering subcutaneous injection and confirm patient self management of injection/referral to district nurse.	C!<br>C2<br>HWB7	4<br>4<br>4	a,b,c,e,f<br>e,h<br>a,b,c,d,g,h
1.8<br>Practitioner can demonstrate the correct completion of appropriate documentation and ensures patient held records are completed.<br>Documents include:<br>• Patient demographics.<br>• Incidental pulmonary embolism symptom chart.<br>• Pulmonary embolism severity index score (PESI).<br>• Incidental pulmonary embolism management data sheet.<br>• History checklist.<br>• Dalteparin for outpatient treatment of pulmonary embolism – GP letter	IK1	1	a,b,c,d,e
1.9<br>Practitioner can describe the care and management of cancer patients with recurrent VTE.	C1<br>C2<br>HWB2<br>HWB5<br>HWB7	4<br>4<br>4<br>4<br>4	a,b,c,d,e,f<br>a,b,c<br>a,b,c,d,e,f,g,h<br>a,b,c,d,e,f,g<br>a,b,c,d,e,f,g

Copyright © Hull and East Yorkshire Hospitals NHS Trust 2012

Practitioner Workbook for Registered Practitioners, Band 7

Introduction

The objective of this document is to develop competent, confident Band 7 practitioners with advanced skills in assessing patients with incidental PE/VTE.

In line with evidence based practice, this workbook has been designed for the practitioner to develop competence with regard to the assessment and management of Oncology/Haematology patients with incidental Pulmonary Embolism and Venous Thromboembolism.

Practitioners can match theory and practice in line with the Trust's standards of care, ensuring quality in care is addressed whilst promoting safe and competent practice.

The competencies have been mapped to STEPS 2 and the Knowledge and Skills Framework and will ensure consistency in practice
The practitioner will undertake in-house theoretical training and practical supervision provided by Dr Maraveyas, Consultant Medical Oncologist, as well as collating evidence to complete this workbook.

The facilitator will support the practitioner through the period of training. Both must agree when competence and confidence are reached and the desired criteria are met.

Practitioners should demonstrate through documented evidence and by working through the accompanying workbook that they are competent.

This role should only be undertaken by Specialist practitioners who have undertaken the relevant training and who have been assessed as competent.

Copyright © Hull and East Yorkshire Hospitals NHS Trust 2012

Question 1

Outline your understanding of your own accountability when carrying out the assessment and management of Oncology/Haematology patients with incidental Pulmonary Embolism and Venous Thromboembolism.

Copyright © Hull and East Yorkshire Hospitals NHS Trust 2012

Question 2

Describe the process of coagulation.

Copyright © Hull and East Yorkshire Hospitals NHS Trust 2012

Question 3

Define the following terms:

a. Thrombocytopenia.

b. Heparin Induced Thrombocytopenia (HIT).

c. Pulmonary Embolism Severity Index (PESI) score.

Copyright © Hull and East Yorkshire Hospitals NHS Trust 2012

Question 4

Describe the signs and symptoms of Heparin Induced Thrombocytopenia (HIT).

Question 5

Describe the management of Heparin Induced Thrombocytopenia (HIT).

Copyright © Hull and East Yorkshire Hospitals NHS Trust 2012

Question 6

Pathology investigations are essential to assessing and managing incidental PE/VTE.

Document those investigations required prior to commencing treatment.

	Normal Range	
FBC		
PT		
PTT		
Clotting screen		
D-Dimer		

Question 7

What are the implications of a persistent elevated D-Dimer result?

Copyright © Hull and East Yorkshire Hospitals NHS Trust 2012

Question 8

Complete a data sheet for Dalteparin Sodium Solution

Date<br>Drug Name Trade Name	
Classification	
Action	
Reason for use	
Methods of Administration	
Describe the toxicities associated with low molecular weight heparins.	

Copyright © Hull and East Yorkshire Hospitals NHS Trust 2012

Question 9

Describe the care and management of patients receiving low molecular weight heparins.

Copyright © Hull and East Yorkshire Hospitals NHS Trust 2012

Question 10

Discuss the following:

What is acute haemorrhage?
What are the signs and symptoms of haemorrhage?
What abnormalities would you expect to see in the patients' blood results?

Copyright © Hull and East Yorkshire Hospitals NHS Trust 2012

What would the treatment and monitoring of an acute episode of haemorrhage be?
What self care and health promotion information would be appropriate to discuss with the patient? Re: bleeding

Copyright © Hull and East Yorkshire Hospitals NHS Trust 2012

Step 2 Practice	Core Competencies<br>Incidental Pulmonary Embolism and Venous Thromboembolism

Question 11

Describe the care and management of cancer patients with recurrent VTE.

Copyright © Hull and East Yorkshire Hospitals NHS Trust 2012

References

British National Formulary: 2007, British National Formulary 54
http://www.bnf.org/bnf/bnf/54/59028.htm#this

Department of Health: 2000 The NHS plan, http://www.dh.gov.uk

Department of Health: 2006, Our Health, Our Care, Our Say.
http://www.dh.gov.uk/en/Policyandguidance/Organisationpolicy/Modernisation/Ourhealthourcareoursay/index.htm

The Case for Commissioning an Incidental PE Pathway – A. Maraveyas, G. Avery & M. Johnson.Thrombosis, (2006). PE and the benefits of LMWH. Vol 10, 4.

HEY Policy No. CP 016 Consent to Examination or Treatment.

HEY Policy No. CP 26 Drug Policy.

Copyright © Hull and East Yorkshire Hospitals NHS Trust 2012

Copyright © Hull and East Yorkshire Hospitals NHS Trust 2011
