# Supplementary material for: Developing a complex intervention for the outpatient management of incidentally diagnosed pulmonary embolism in cancer patients
Source: BMC Health Serv Res. 2013 Jun 27;13:235. doi: 10.1186/1472-6963-13-235 (PMC3718646; doi:10.1186/1472-6963-13-235)
Supplement: Additional file 3 — Demographics and management data-sheets, patient symptom questionnaire. [file 1472-6963-13-235-S3.pdf]

HULL AND EAST YORSHIRE HOSPITALS NHS TRUST

DEPARTMENT OF CLINICAL ONCOLOGY & HAEMATOLOGY

Initial Assessment	
Patient ID Label<br><br><br>Telephone:	Next of Kin/ Carer:<br>Name:<br>Address:<br>Telephone:
Presenting History/ Diagnosis/Treatment	
Past Medical History	
Any Allergies	
Regular Medication/ PRN	
General Practitioner	
Name:<br>Address:<br><br>Telephone Number:	
Observations	
Temperature -	
Pulse -	
Respirations -	
Blood Pressure -	
O² saturation -	
Weight –	
Performance Status PS	
Patient Follow up	
Patient consents to future telephone follow up calls.<br><br>Patients Signature……………………………………… Date…………………………<br><br>Print Name………………………………………………	
Assessed By: Band: Date	

Chemotherapy Nurse Specialist Team
Reviewed 11/12

Communication Sheet

PATIENTS NAME		
UNIT NUMBER		
DATE/TIME	ADDITIONAL INFORMATION	SIGNATURE/GRADE/TITLE

INCIDENTAL PULMONARY EMBOLISM MANAGEMENT DATA-SHEET

**STEP1**	PATIENT DETAILS

Date of CT scan: …...........
Date patient seen: …...........
Patient Demographics: (Affix Label)

- Contact numbers
- GP
- Date of last anti-cancer treatment.........
- List of medications (see below)
- Performance status……………………
- Site of PE/VTE……………………………
- PE Breakdown – Bilateral…………………Yes/no
  Central…………………..Yes/no
  Segmental………………Yes/no
  Subsegmental……………Yes/no
  Other……………………………

**STEP 2**	RECORD COURSE OF ACTION	PESI SCORE=

A) ***PESI ≤ 124 with O_2 Sat> 90%, PR <110, RR<30 and Syst BP>100 mmHg***

- Manage as outpatient
- Contact DVT clinic **Contacted YES/NO**
- Issue Anticoagulation Pack after demonstrating correct injection technique. Check pack includes demonstration DVD and written advice to patient.
  Issued Yes/No
- Book clinic follow-up appointment **Date.................**
- Issue standard GP letter
  - **If in any doubt or cardio-respiratory parameters abnormal** seek senior clinical advice
    - Admitted by Clinician-Complete B

B) ***PESI >124 - Admission may be necessary***

- Seek clinical advice **Clinician...............**
- Admitted to Ward...................
- Discharged by Clinician **Yes/No**
  - (complete A)

C) Patient already in-patient on WD…………..

- Treatment LMWH **Yes/No**
- Treatment changed **Yes/No**

**STEP 3**	ADMINISTRATION

- Please affix Demographics label on supplementary sheet
- Please arrange for notes, this sheet & supplementary sheet to go to data co-ordinator:
  Ext............... FAX................ E-mail..............

Hull PCT patients to Westbourne Ave: Tel No...
East Riding of Yorkshire PCT patients to............................. (Contact relevant practice)

Please list patient medications:

INCIDENTAL PULMONARY EMBOLISM SYMPTOM CHART
(Patient to fill in – Please help if necessary)

'General Questions'

Please answer all the questions

1) Have you ANY new symptoms above the usual symptoms related to your cancer or the treatment? YES/NO
If YES please give details, if NO go to next question

Symptom(s):

Date symptoms started (you do not have to be exact):

If you are on chemotherapy and you can relate the start of these symptoms to any specific cycle of treatment please record the cycle number here (you may have the chemotherapy information in your 'Red Book')

CYCLE…….

2) Have any of your USUAL symptoms suddenly worsened? YES/NO
If YES please give details, if NO go to 'Specific Questions'

Symptom(s):

Date symptoms suddenly worsened (you do not have to be exact):

If you are on chemotherapy, and you can relate the worsening of these symptoms to any specific cycle of treatment please record the cycle number here (you may have the chemotherapy information in your 'Red Book')

CYCLE………

'Specific Questions'

**1) <u>Are you short of breath?</u>**	**YES**	**NO**
	If NO go to question 2	
a. Is this new?	Yes	No
b. If it's been there before has it worsened?	Yes	No

**2) <u>Do you have fatigue?</u>**	**YES**	**NO**
	If NO go to question 3	
a. Is this new?	Yes	No
b. If it's been there before has it worsened?	Yes	No
**3) <u>Do you have chest pain?</u>**	**YES**	**NO**
	If NO go to question 4	
a. Is this new?	Yes	No
b. If it's been there before has it worsened?	Yes	No
**4) <u>Do you have leg swelling?</u>**	**YES**	**NO**
	If NO go to question 5	
a. Is this new?	Yes	NO
b. If it's been there before has it worsened?	Yes	No
**5) <u>Have you coughed blood?</u>**	**YES**	**NO**
	If NO please hand back to Doctor, or nurse	
a. Is this new?	Yes	No
b. If it's been there before has it worsened?	Yes	No

Thank you for taking the time to fill in this questionnaire. Please hand it back to the doctor, or nurse who is attending you.

Date………………………………………

Signature………………………………..
