# Supplementary material for: Developing a complex intervention for the outpatient management of incidentally diagnosed pulmonary embolism in cancer patients
Source: BMC Health Serv Res. 2013 Jun 27;13:235. doi: 10.1186/1472-6963-13-235 (PMC3718646; doi:10.1186/1472-6963-13-235)

PATHWAY 1 – Cancer Patients

Unsuspected PE/VTE Identified at Time of Scanning
Monday to Friday 9.00a.m. – 5.00p.m.

Radiographer suspects PE on reviewing images.

↓

Radiographer discusses with Radiologist and PE confirmed.

↓

Radiologist/Radiographer informs the Chemotherapy Nurse Specialist Team on 761082.

↓

If patient is **asymptomatic,** they should be allowed home and informed that the Chemotherapy Nurse Specialist Team will be contacting them to arrange assessment in Oncology outpatients.

If patient is symptomatic or unwell, they should be admitted to the Queens Centre via Bleep 500.

↓

If unable to contact Chemotherapy Nurse Specialist Team, message to be left on answerphone.

Radiologist informs patient. Sends patient to Oncology outpatients.

↓

Radiologist reports scan and arranges immediate typing.
Report then faxed to Oncology secretaries on 607739.
Result available to view on Patientcentre.

↓

Radiologist/Radiographer completes Data Collection Form and sends to Clinical Trials Officer, CT/MRI Suite CHH.

<u>PATHWAY 2 – Cancer patients</u>

Unsuspected PE/VTE Identified at Time of Reporting
Monday – Friday 9.00 a.m. – 5.00 p.m.

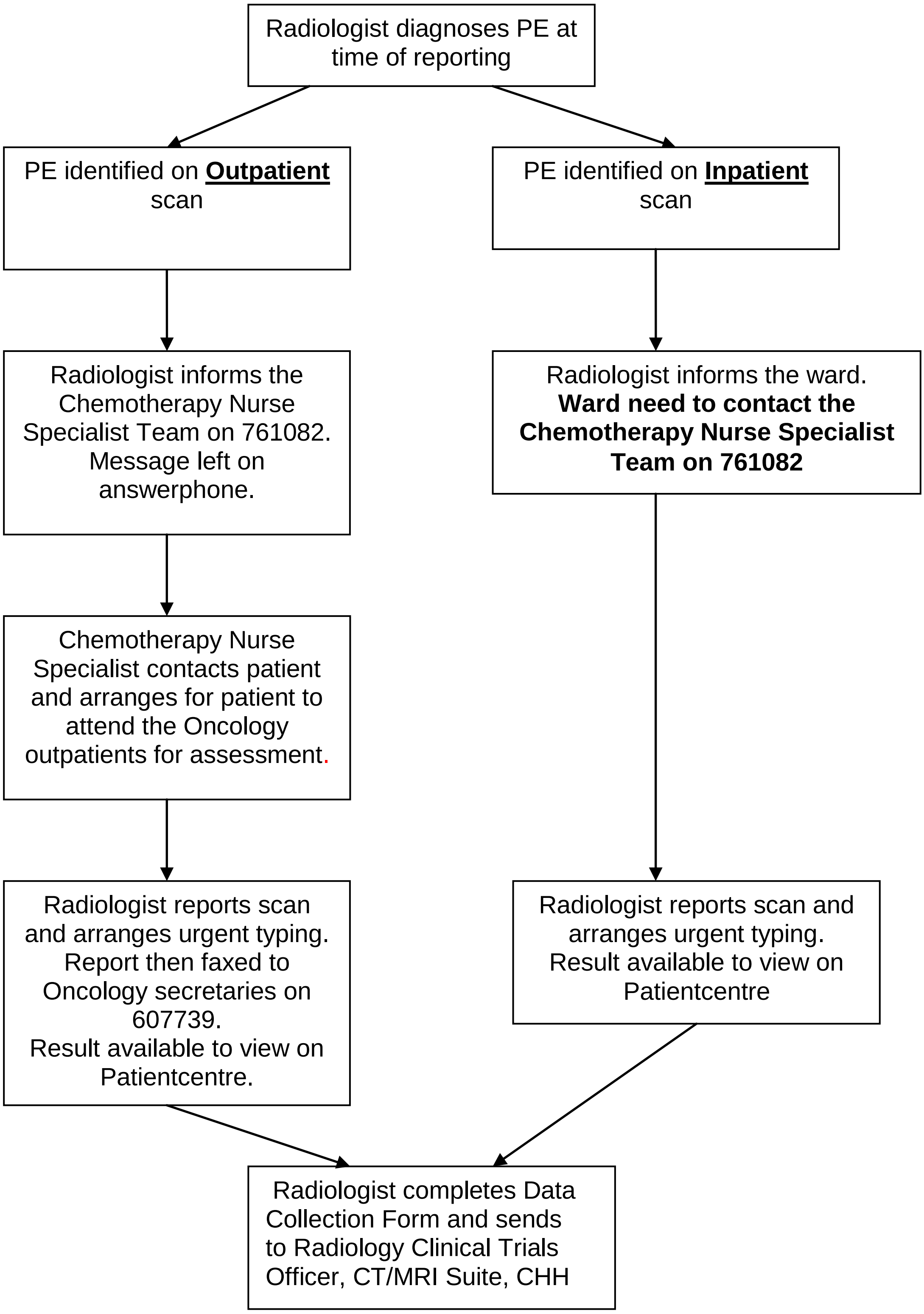

PATHWAY 3 – Cancer Patients

Unsuspected PE/VTE Identified at reporting out of hours Monday to Friday 5.00 p.m. – 8.00 a.m. Saturday and Sunday

Radiologist diagnoses PE at time of reporting

PE identified on **Outpatient** scan

Radiologist informs the Chemotherapy Nurse Specialist Team on 761082 Message left on answerphone

Radiologist reports scan and arranges urgent typing next working day. Report then faxed to Oncology secretaries on 607739. Result available to view on Patientcentre.

Chemotherapy Nurse Specialist Team contacts patient and arranges for patient to attend the Oncology outpatients for assessment.

PE identified on **Inpatient** scan

Radiologist informs the ward. **Ward need to contact the Chemotherapy Nurse Specialist Team on 761082**

Radiologist reports scan and arranges urgent typing next working day. Result available to view on Patientcentre

Radiologist completes Data collection Form and sends to Radiology Clinical Trials Officer, CT/MRI Suite, CHH

PATHWAY 4 – Cancer Patients

Unsuspected PE/VTE Identified at Time of Scanning Out Of Hours (Monday to Friday 5.00 p.m. – 8.00 a.m. Saturday and Sunday)

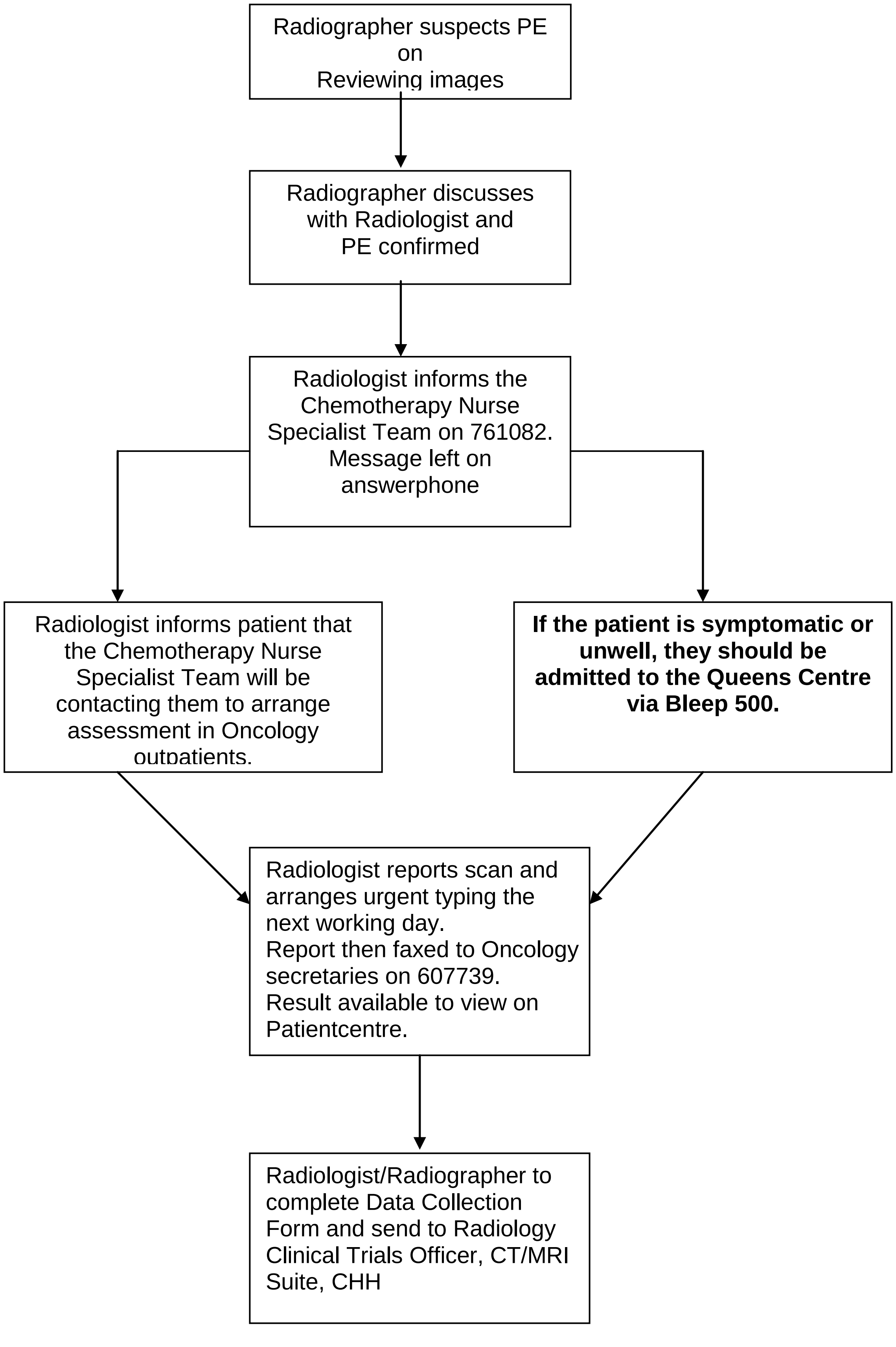

Supplement: Additional file 4 — Notification/referral pathways. [file 1472-6963-13-235-S4.pdf]
